# Retraction Note: The shadow economy in South Asia: dynamic effects on clean energy consumption and environmental pollution

**DOI:** 10.1007/s11356-024-32890-1

**Published:** 2024-03-14

**Authors:** Muhammad Tayyab Sohail, Sana Ullah, Muhammad Tariq Majeed, Ahmed Usman, Zubaria Andlib

**Affiliations:** 1 School of Public Administration, Xiangtan University, Xiangtan, Hunan China; 2 School of Economics, Quaid-i-Azam University, Islamabad, Pakistan; 3 Department of Economics, Government College University Faisalabad, Faisalabad, Pakistan; 4 School of Economics, Federal Urdu University, Islamabad, Pakistan

**Retraction Note: Environmental Science and Pollution Research**

10.1007/s11356-021-12690-7

The Publisher has retracted this article in agreement with the Editor-in-Chief. An investigation by the publisher found a number of articles, including this one, with a number of concerns, including but not limited to compromised peer review process, inappropriate or irrelevant references, containing nonstandard phrases or not being in scope of the journal. Based on the investigation's findings the publisher, in consultation with the Editor-in-Chief therefore no longer has confidence in the results and conclusions of this article.

The authors disagree with this retraction.

